# Peripheral veno-arterial extracorporeal membrane oxygenation as a bridge to surgery in type A aortic dissection: a review on strategic approach to managing malperfusion syndrome

**DOI:** 10.1051/ject/2025015

**Published:** 2025-09-15

**Authors:** Ignazio Condello

**Affiliations:** 1 Department of Cardiac Surgery, Anthea Hospital, GVM Care & Research, Anthea Hospital, University of Insubria Varese Via Camillo Rosalba 35/37 70124 Bari Italy

**Keywords:** Acute type A aortic dissection, Malperfusion syndrome, ECMO, Emergency, Cardiovascular care, Patient survival

## Abstract

*Background*: Acute Type A aortic dissection (ATAAD) is a critical cardiovascular emergency characterized by high mortality rates and complex management challenges. The presence of a tear in the ascending aorta often extends into the aortic arch and descending thoracic aorta, leading to malperfusion syndrome, a severe condition resulting from obstructed blood flow to vital organs. Despite the high risks associated with ATAAD, the use of Peripheral Veno-Arterial (VA), Extracorporeal Membrane Oxygenation (ECMO) remains controversial. This intervention aims to maintain systemic circulation and organ perfusion, potentially stabilizing patients prior to surgical repair. *Materials and methods*: A narrative review of the literature was conducted through a comprehensive search of PubMed and Embase databases, covering the period from January 2000 to March 2025. Keywords included “ECMO”, “Type A Aortic Dissection”, “malperfusion”, and “bridge to surgery”, among others. Although this is a narrative review, the methodology was guided by the PRISMA guidelines to ensure transparency and reproducibility in the selection and reporting of the included studies. *Results*: Ten relevant articles were identified, including observational studies, case series, and reviews. This narrative review presents the role of peripheral VA ECMO in managing ATAAD, focusing on the timing of ECMO initiation and its implications for patient outcomes. The approach emphasizes rapid deployment following confirmation that the iliac and femoral arteries are free from dissection involvement, ensuring safe cannulation and effective circulatory support. The discussion also explores ECMO’s role as a bridge to surgery, detailing its impact on preventing malperfusion to critical organs such as the brain, kidneys, and splanchnic organs. *Conclusion*: While VA ECMO offers a potential lifeline for patients with severe ATAAD, its application must be carefully considered within an integrated treatment strategy. The ongoing debate and emerging research underscore the need for further studies to define clear guidelines and optimize ECMO’s use in this high-risk patient population. The balance between preventing malperfusion and managing increased myocardial workload presents a complex clinical challenge, necessitating continued investigation and dialogue within the medical community.

## Background

Acute Type A aortic dissection (ATAAD) represents one of the most severe cardiovascular emergencies due to its high mortality rate and complex management challenges. This condition involves a tear in the ascending aorta, which can extend to the aortic arch and descending thoracic aorta, creating a false lumen that compromises blood flow and can lead to catastrophic outcomes, including malperfusion syndrome. Malperfusion syndrome, a critical complication of ATAAD, results when the dissection flap obstructs blood flow to major arterial branches, leading to ischemia of vital organs. According to a nationwide analysis by Goel et al., preoperative malperfusion occurs in approximately 27.7% of ATAAD cases, significantly increasing operative mortality rates. The study underscores that malperfusion most frequently affects the extremities, kidneys, and brain, and varies in mortality impact based on the region affected [1]. For instance, coronary and mesenteric malperfusions are associated with the highest mortality risks. Despite the acute risks associated with ATAAD and its complications, the use of Peripheral Veno-Arterial (VA) Extracorporeal Membrane Oxygenation (ECMO) remains a debated intervention. VA ECMO serves to maintain systemic circulation and vital organ perfusion, potentially stabilizing patients prior to surgical repair. This approach could be useful for peripheral centers that do not have cardiac surgery programs, and the expected transfer transportation time is greater than 15 min [2]. However, as reviewed by Capoccia and Maybauer, literature on the effectiveness of VA ECMO in this specific setting is scant and mixed, with studies indicating both potential benefits and significant challenges. Their systematic review reveals no conclusive evidence for or against ECMO’s routine use in major aortic surgery but suggests its cautious application might be justified, particularly in refractory cases where conventional therapies fail [2]. The controversial nature of ECMO’s role in ATAAD treatment is further complicated by technical considerations and the variability in outcomes associated with its use. For example, peripheral VA ECMO may increase left ventricular afterload, which requires careful management to optimize beneficial effects and minimize potential harm. The combined use of VA ECMO with other mechanical support devices like an Intra-Aortic Balloon Pump (IABP) or Impella has been explored as a strategy to reduce mortality, suggesting a multifaceted approach might be necessary for optimal patient management [3, 4]. While VA ECMO offers a potential lifeline for patients with severe ATAAD and malperfusion syndrome, its application should be carefully considered within the context of an integrated treatment strategy tailored to individual patient conditions.

## Materials and methods

This narrative review was conducted to explore the role of peripheral veno-arterial extracorporeal membrane oxygenation (VA ECMO) in the management of acute Type A aortic dissection (ATAAD), with a specific focus on malperfusion syndrome and its potential application as a bridge to surgery. A comprehensive literature search was performed using the PubMed and Embase databases, covering publications from January 2000 to March 2025. The search strategy included a combination of free-text terms and Medical Subject Headings (MeSH) such as: “ECMO” *OR* “Extracorporeal Membrane Oxygenation”*,* “Type A Aortic Dissection”*,* “Acute Aortic Syndrome”*,* “malperfusion*”*, *“*bridge to surgery”*,* “hemodynamic support”*, and* “organ protection”. No language restrictions were applied, provided that an abstract was available in English. Although this is a narrative review, the methodology was informed by the PRISMA (Preferred Reporting Items for Systematic Reviews and Meta-Analyses) guidelines. The aim was to integrate current evidence into a reasoned proposal for its application as a supportive strategy in selected ATAAD cases.

## Results

A total of 10 relevant articles were identified and selected for inclusion. These consisted of observational studies, case series, systematic reviews, and technical reports that addressed the use of VA ECMO in the setting of ATAAD. The initiation of Peripheral VA ECMO in patients diagnosed with acute ATAAD represents a critical decision point that impacts the overall outcome. The primary goal is to establish ECMO support as quickly as possible following the diagnosis, provided that comprehensive imaging confirms the absence of iliac and femoral artery involvement in the dissection. This ensures that the chosen cannulation sites are safe and that the ECMO can provide effective circulatory support without exacerbating the dissection.

1. *Imaging and Assessment*: Before VA ECMO is initiated, a detailed assessment through imaging, typically a CT angiography, is essential. This imaging must confirm that the iliac and femoral arteries, common sites for ECMO cannulation, are free from involvement by the dissection. The integrity of these vessels is crucial, as cannulation in a dissected vessel can lead to catastrophic complications, including extension of the dissection or complete vessel rupture [2].
2. *Rapid Deployment*: Once safe cannulation pathways are confirmed, the rapid deployment of ECMO is vital. The window from diagnosis to ECMO initiation is a critical period where the patient’s condition can deteriorate quickly. Fast and effective ECMO deployment helps stabilize the patient’s hemodynamics, providing a bridge to surgical intervention while preventing end-organ damage due to decreased perfusion [5].
3. *Preventive Strategy*: The use of ECMO in ATAAD is not only about immediate survival but also about preventing further complications. By maintaining systemic circulation and oxygenation, ECMO helps prevent malperfusion to vital organs, which may not yet be symptomatic but could fail rapidly if blood flow is not restored or supported [6].
4. *Coordination of Care*: Implementing ECMO requires coordinated care between multiple ECMO teams, including perfusionists, cardiothoracic surgery, cardiology, critical care, and vascular surgery [5]. This multidisciplinary approach is essential for ensuring that ECMO is initiated safely, timely, and effectively and that the patient is rapidly transitioned to definitive surgical repair as soon as they are stabilized [7].

## Discussion

The period from Peripheral VA ECMO initiation to surgery represents a critical window that must be managed with precision. Rapid deployment of ECMO followed by swift surgical intervention is essential to minimize risks such as bleeding and thrombotic complications associated with prolonged ECMO support [2]. Importantly, the quick transition to surgery is not only a matter of reducing ECMO-related complications but is also crucial for addressing the root cause of the dissection before significant anatomical and physiological deterioration occurs [7, 8]. Moreover, peripheral ECMO can increase myocardial workload, which necessitates a careful balance between preventing malperfusion and avoiding excessive cardiac strain. In the setting of ATAAD, where the heart may already be under considerable stress due to compromised aortic integrity and altered hemodynamics, ECMO provides critical support by mechanically offloading the heart [9].

### VA ECMO as a bridge to surgery and malperfusion prevention

VA ECMO serves an indispensable role in the management of ATAAD by providing hemodynamic stability and maintaining organ perfusion. This is crucial in the pre-operative phase to prevent malperfusion syndrome, which can severely impair renal, cerebral, and splanchnic organ function.

1. *Cerebral Protection*: The brain is highly susceptible to ischemia resulting from compromised blood flow in ATAAD. ECMO facilitates cerebral protection by ensuring adequate cerebral perfusion. This is achieved through the maintenance of systemic blood pressure, which is critical for preventing cerebral ischemia and subsequent neurological deficits. The precise control of blood flow provided by ECMO helps avoid the drastic fluctuations in blood pressure that can exacerbate brain injury, thus safeguarding neurologic function during this vulnerable period [1].
2. *Renal Protection*: Renal function can deteriorate rapidly under conditions of low perfusion characteristic of ATAAD. ECMO supports renal function by ensuring continuous renal blood flow. This is vital for filtering metabolic wastes and managing fluid balance, which is critical in preventing acute kidney injury – a common complication that can significantly extend hospital stays and affect post-operative recovery [2].
3. *Protection of Splanchnic Organs*: Splanchnic organs, such as the intestines and liver, require a consistent and high-level blood supply to maintain function. ECMO supports these organs by ensuring adequate blood flow, thus preventing ischemic injuries that could lead to severe complications like mesenteric ischemia. By providing a stable flow and preventing fluctuations in blood pressure, ECMO helps maintain the integrity and function of the gastrointestinal tract and liver, which are crucial for recovery and overall health [1].

### Challenges of implementing VA ECMO in ATAAD

Despite its significant benefits, implementing VA ECMO in ATAAD is fraught with challenges that require meticulous management:

- *Cannulation Challenges*: Proper cannulation is paramount and must be performed with precision to avoid damaging the vessels, particularly in a dissection scenario where the integrity of vascular structures may be compromised. The choice of cannulation sites requires careful consideration and ultrasound-guided techniques may be employed to enhance safety and accuracy. Correct positioning of the cannulas is crucial not only to ensure effective ECMO function but also to minimize the risk of complications such as limb ischemia, vascular injury, and improper perfusion [4].
- *Management of Hemodynamics*: Balancing the hemodynamic support provided by ECMO without exacerbating the heart’s workload, especially the left ventricle, necessitates careful adjustment and continuous monitoring [10]. The aim is to ensure enough perfusion pressure to prevent organ malperfusion while avoiding excessive afterload that could strain the heart, particularly when left ventricular function is already compromised [3]. Echocardiographic monitoring becomes a valuable tool in this context, providing real-time feedback on cardiac function and ECMO impact [10]. While specific targets vary, typical mean arterial pressures ≥65 mmHg and flows to match 60–80 mL/kg/min are often referenced, the role of echocardiography is fundamental in titrating these settings [2], but further studies are needed to validate these target parameters. This allows for the timely adjustment of ECMO flow and pressure settings to optimize cardiac unloading and minimize myocardial stress.
- *Systemic Anticoagulation*: The need for anticoagulation with ECMO introduces significant risks of bleeding, particularly in the surgical setting where patients are already at a heightened risk due to the invasive nature of their conditions. Managing these risks while maintaining effective anticoagulation to prevent clotting within the ECMO circuit requires a delicate balance. This often involves continuous monitoring of coagulation parameters and individualized adjustments to anticoagulation protocols [2]. While there are currently no standardized dosage recommendations tailored specifically to VA ECMO use in the setting of acute type A aortic dissection (ATAAD), particularly when employed as a bridge to surgery, aligning clinical decisions with established guidelines and the standard of care remains essential. Following best practice protocols ensures consistency, safety, and efficacy in patient management even in areas where clinical evidence is still evolving. By adhering to expert consensus and institutional protocols derived from broader ECMO experience, clinicians can provide care that is both evidence-informed and adaptable to the nuances of ATAAD. This approach not only supports optimal patient outcomes but also fosters a framework for ongoing evaluation and refinement of ECMO strategies in this complex and high-risk population [5].

## Conclusion

The use of Peripheral VA ECMO in the management of acute ATAAD presents a critical yet debated intervention within cardiovascular care. While ECMO is instrumental in preventing malperfusion and safeguarding organ function, its impact on increasing the myocardial workload invites a complex balance that remains a subject of clinical scrutiny. This juxtaposition underscores ECMO not only as a lifesaving measure but also as a catalyst for ongoing debate regarding its optimal application. Given the dual-edged nature of ECMO, offering profound benefits in preventing organ failure while potentially exacerbating cardiac stress, it is clear that this therapeutic strategy occupies a vital space in the continuum of care for ATAAD. However, the conversation about ECMO’s role does not end here. It is indeed an open perspective that demands further exploration and understanding. The complexity of ECMO’s effects on cardiac dynamics, particularly in the context of peripheral cannulation and its associated afterload implications, warrants deeper investigation. Future studies are required to delineate clear guidelines and develop advanced protocols that not only maximize the benefits of ECMO but also mitigate its risks. Continued research is essential to refine ECMO deployment strategies, enhance real-time monitoring and management techniques, and ultimately, to ensure that this critical care tool can be used to its full potential without undue harm.

## Data Availability

No new data were created or analyzed in this study. Data sharing is not applicable to this article.
